# Effects of lncRNA HOXA11-AS on Sevoflurane-Induced Neuronal Apoptosis and Inflammatory Responses by Regulating miR-98-5p/EphA4

**DOI:** 10.1155/2023/7750134

**Published:** 2023-04-05

**Authors:** Li Zhao, Zhonghui Wang, Haitao Chen, Yaxi Du, Weihao Ma, Qunfen Tao, Xiang Ma, Zeming Wu, Jing Peng

**Affiliations:** ^1^Department of Anesthesiology, The Third Affiliated Hospital of Kunming Medical University (Yunnan Cancer Hospital), Kunming, 650118 Yunnan, China; ^2^Department of Ultrasound, The Third Affiliated Hospital of Kunming Medical University (Yunnan Cancer Hospital), Kunming, 650118 Yunnan, China; ^3^Department of Molecular Diagnosis Center, The Third Affiliated Hospital of Kunming Medical University (Yunnan Cancer Hospital), Kunming, 650118 Yunnan, China; ^4^Department of Operation Room, The Third Affiliated Hospital of Kunming Medical University (Yunnan Cancer Hospital), Yunnan, China

## Abstract

**Objective:**

To explore the molecular mechanism of sevoflurane-induced neurotoxicity and to determine whether lncRNA HOXA11-AS affects sevoflurane-induced neuronal apoptosis and inflammation by regulating miR-98-5p/EphA4.

**Methods:**

Morris water maze (MWM) test was used to detect the learning and memory ability of rats, HE staining was used to observe hippocampal pathology, TUNEL staining was used to detect the level of neuronal apoptosis, and RT-qPCR was used to detect the expression of HOXA11-AS, miR-98-5p, IL-6, IL-1*β*, and TNF-*α*. At the same time, the contents of IL-6, IL-1*β*, and TNF-*α* in serum were detected by ELISA. The expressions of apoptosis-related proteins EphA4, Bax, Cleaved caspase 3, and Bcl-2 were detected by Western blot. The dual-luciferase gene reporter verified the targeting relationship between HOXA11-AS and miR-98-5p and the targeting relationship between miR-98-5p and EphA4.

**Results:**

The expression of HOXA11-AS was observed in sevoflurane-treated rats or cells and promoted neuronal apoptosis and inflammation. HOXA11-AS was knocked out alone, or miR-98-5p was overexpressed which attenuates neuronal apoptosis and inflammatory inflammation after sevoflurane treatment. Furthermore, knockdown of HOXA11-AS alone was partially restored by knockdown of miR-98-5p or overexpression of EphA4.

**Conclusion:**

Inhibition of lncRNA HOXA11-AS attenuates sevoflurane-induced neuronal apoptosis and inflammatory responses via miR-98-5p/EphA4.

## 1. Introduction

Each year, millions of young children are given general anesthesia to facilitate surgical operations and examinations, and sevoflurane is one of the most common inhalation anesthetics for children [1, 2]. Although general anesthesia has traditionally been considered safe and reversible in adults, however, recent preclinical studies have shown that exposure of the immature brain to sevoflurane in the neonatal period may induce neuronal apoptosis, synaptic deficits, neuroinflammation, tau phosphorylation, and other insults, leading to ultimately impair learning and memory abilities [1, 3, 4]. However, the influence of sevoflurane on brain development is unclear. Therefore, mechanistic studies of neurotoxicity caused by sevoflurane are significant and find an effective method to improve neurobehavioral dysfunction caused by general anesthesia.

Studies have found that nerve cell apoptosis and inflammation are the main mechanisms of cognitive impairment caused by sevoflurane [5, 6]. Homeobox A11 antisense (HOXA11-AS) is an lncRNA, located on the HOX gene cluster. The current research on HOXA11-AS mainly focuses on cancer. It was found that upexpression of HOXA11-AS appeared in a variety of cancers, which was usually related to poor prognosis.

It is found that HOXA11-AS is upregulated in multiple cancers and is usually associated with poor prognosis [7]. So far, HOXA11-AS has also been found to play a role in neuroinflammation and neuroapoptosis. For example, Li et al. [8] found that HOXA11-AS aggravates neuroinflammation caused by microglia after traumatic brain injury. Cao et al. [9] found that inhibiting HOXA11-AS ameliorated neuroinflammation and neuronal apoptosis in MPTP-induced Parkinson's mouse models. However, whether HOXA11-AS is involved in nerve cell apoptosis and inflammation caused by sevoflurane is still unclear.

As a member of the let-7 family, miR-98-5p is associated with oxidative stress, cell apoptosis, and survival in many crucial biological processes of various cells [10, 11]. Sun et al. [10] showed that miR-98-5p could ameliorate neuronal damage induced by oxygen glucose deprivation/reoxygenation (OGD/R). Upregulation of miR-98-5p alleviated sevoflurane-induced social and emotional disorders in neonatal mice [12]. EphA4 receptors regulate neurogenesis and border formation in the developing brain [13], and the absence of EphA4 improves neuroinflammation and tissue damage induced by traumatic brain injury [14]. In addition, studies have shown that downregulating the expression level of EphA4 in developing neurons can reduce propofol-induced neurotoxicity [15]. However, the role of EphA4 in the neurotoxicity caused by sevoflurane has not been discussed yet.

Therefore, this study will investigate whether HOXA11-AS affects neuronal apoptosis and inflammation induced by sevoflurane via miR-98-5p/EphA4.

## 2. Materials and Methods

### 2.1. Animal Experiment

There are a total of 30 Sprague Dawley (SD) rats (7 days old and weighing 180-200 g; Sibf (Beijing) Biotechnology Co., Ltd., China). Rats were fed for 7 days under standard laboratory conditions (12 : 12 alternating cycle of light and dark, 22°C, and 60% relative humidity) and divided into 3 groups randomly (10 rats per group): normal control (NC) group (exposed to air), sevoflurane group (rats were continuously given 2.2% sevoflurane in 21% oxygen for 3 days, 2 h per day), or si-HOXA11-AS+sevoflurane group (the si-HOXA11-AS interference lentiviral vector was injected into the bilateral hippocampus immediately following the first dose of anaesthesia). All rats returned to the cage after recovery. No deaths occurred during or within two weeks of dosing. After 3 days of anesthesia, four mice from each group were euthanized, and hippocampus was collected for follow-up analysis. The rest of the rats were used for analysis of cognitive impairment. All animal experiments were approved by the ethics committee of the Third Affiliated Hospital of Kunming Medical University and follow the National Institutes of Health Laboratory Animal Care and Use Guidelines.

### 2.2. Real-Time Quantitative PCR (qRT-PCR)

Extract total RNA from cell tissues using Trizol kit, and then, reverse transcribed into cDNA with PrimeScript™ RT kit (Takara, Shiga, Japan). Use the SYBR®Premix Ex TaqTM II kit (RR820A, Takara) to perform real-time fluorescent qPCR on a fluorescent qPCR device. Finally, U6 or *β*-actin was selected as internal reference, after amplification 2^−ΔΔCt^ method was applied to calculate the relative expression of HOXA11-AS and miR-98-5p. The primer sequences are shown in Table 1.

### 2.3. Morris Water Maze (MWM) Test

The MWM test was conducted by an operator who was unaware of the treatment group. The cylindrical pool, 150 cm in diameter and 60 cm in depth, was divided into four identical quadrants and filled with water up to depth of 30 cm (25°C ± 1°C), then a platform (10 cm in diameter) was hidden 1 cm underwater. After sevoflurane anesthesia for 2 weeks, the rats were placed in the maze from different locations (north, south, east, or west), allowing them to find the hidden platform within 60 s and stay on it for 15 seconds. If a rat fails to find the platform within 60 s, gently guide it to the platform and allow it staying on it for 15 s. The motion detection software (Shanghai Mobile Data Information Technology Co., Ltd., China) was applied for recording the escape latency. The test is carried out 4 times a day for 4 days, and the test interval is 30-40 minutes. On the 5th day, the platform was taken away. The rats were allowed to swim freely for 120 s starting from the opposite quadrant, and crossing platform numbers of rats were recorded simultaneously.

### 2.4. Hematoxylin and Eosin (HE) Stain

The paraffin sections of hippocampal tissues were cut into slices of 4 *μ*m. Sections were deparaffinized and stained with eosin for 1 min following hematoxylin for 3 min. Sections were dehydrated and infiltrated. The pathological changes of hippocampus tissue were observed under light microscope (XP-330, Shanghai Bingyu Optical Instrument Co., Ltd.).

### 2.5. TUNEL Stain

An in situ cell death detection kit (Roche, Indianapolis, in, USA) was used to detect neuronal apoptosis and experimental procedures referring to the manufacturer's instructions. The nuclei were stained with DAPI (2-(4-amidinophenyl)-6-indolecarbamidine dihydrochloride), and TUNEL staining was assessed. It will be perceived as a positive feature if the nucleus were labeled with DAPI and TUNEL. Randomly select 5 areas to observe cell apoptosis.

### 2.6. ELISA

ELISA kits for IL-1*β*, TNF-*α*, and IL-6 were all purchased from Abcam (UK), and the levels of inflammation levels in hippocampal tissue and cell supernatant were detected according to the kit instructions.

### 2.7. Cell Culture and Treatment

HEK-293 T cells were cultured in high-glucose Dulbecco's modified eagle's medium (DMEM, Gibco, USA), which was added 10% fetal bovine serum (FBS, Gibco, USA) and 1% penicillin streptomycin (Gibco, USA) solution. The hippocampus tissue pieces of a 1-day-old SD rat was digested with trypsin. A series of experimental operations including grinding and centrifugation were carried out. The cells were inoculated on a 10 mm culture dish coated with poly-D-lysine (10 mmol/L) at a concentration of 1 × 10^6^ cells per mL and cultured in neurobasal medium (Gibco, USA) containing 2% B27 (Gibco, USA), 2 mM L-glutamine, 50 U/mL penicillin, 50 U/mL streptomycin (Gibco, USA) at 37°C, and 5% CO_2_. After 4 hours of incubation, the medium was changed to serum-free B27/neural basal medium. Replace the medium every 3 days. The cells were induced at 37°C, 5% CO_2_, 21% O_2_, and 4% sevoflurane for 6 h to simulate sevoflurane anesthesia in vitro. The si-HOXA11-AS, miR-98-5p mimic, and OE-EphA4 plasmids were transfected into neurons according to the Lipofectamine 2000 (Invitrogen, USA) instructions. These plasmids were synthesized and constructed by Shanghai GenePharma Co., Ltd. (Shanghai, China). After culturing overnight at 37°C for 48 h, they were used for subsequent experiments.

### 2.8. Western Blot

Protein samples are extracted from tissues and cells using RIPA lysis buffer (Thermo Fisher Scientifc, USA), and its concentration was analyzed with a bicinchoninic acid (BCA) kit (Thermo Fisher Scientific, USA). Protein, separated by SDS-PAGE, was adsorbed by polyvinylidene fluoride (PVDF) and then blocked with 5% nonfat milk at room temperature for an hour. Next, the membranes were incubated with specific primary antibody overnight at 4°C. The following day, membranes were probed with secondary IgG labeling horseradish peroxidasefor 1 h at room temperature. All antibodies were purchased from Abcam PLC, and the antibody concentration control refer to the manual (Bax, 1 : 2000; cleaved caspase-3, 1 : 2000; Bcl-2, 1 : 2000; *β*-actin,1 : 2000; IgG, 1 : 500). The relative gray value of protein bands compared with *β*-actin was read by ImageJ software.

### 2.9. Dual-Luciferase Reporter Assay

The wild-type (WT) and mutant (MUT) reporter plasmids of HOXA11-AS and EphA4 (WT-HOXA11-AS and MUT-HOXA11-AS, WT-EphA4, and MUT-EphA4, designed and provided by GenePharma) were cotransfected with negative control and miR-98-5p mimic into HEK-293T cells. After culturing for 48 h, the luciferase activity was detected by dual-luciferase reporter assay system (Promega, USA).

### 2.10. Immumohistochemical Staining

Paraffin sections of hippocampal tissue were dried overnight at 37°C and deparaffinized. Soak the slices in 0.3% methanol H_2_O_2_ solution. Sections were boiled in 10 mM sodium citrate buffer. Incubate overnight with mouse monoclonal anti-EphA4 (1 : 100,Abcam, UK) at 4°C. Subsequently, incubate with 50 *μ*L secondary antibody (1 : 100, Abcam, UK). The sections were stained with diaminobenzidine, counterstained with hematoxylin, then observed and photographed with optical microscope (Olympus, Japan). The positive rate of neurons above 10% is positive expression.

### 2.11. Statistical Analysis

All data were expressed as mean ± SD. All experiments were repeated at least 3 times. Student's *T* test was used to compare the two groups. The one-way ANOVA and post hoc test were used to compare the multiple groups. SPSS 26 was used for analysis. *P* < 0.05 was considered a statistically significant difference.

## 3. Results

### 3.1. Inhibition of HOXA11-AS Alleviates the Cognitive Impairment Caused by Sevoflurane in Rats

**Figure 1:**
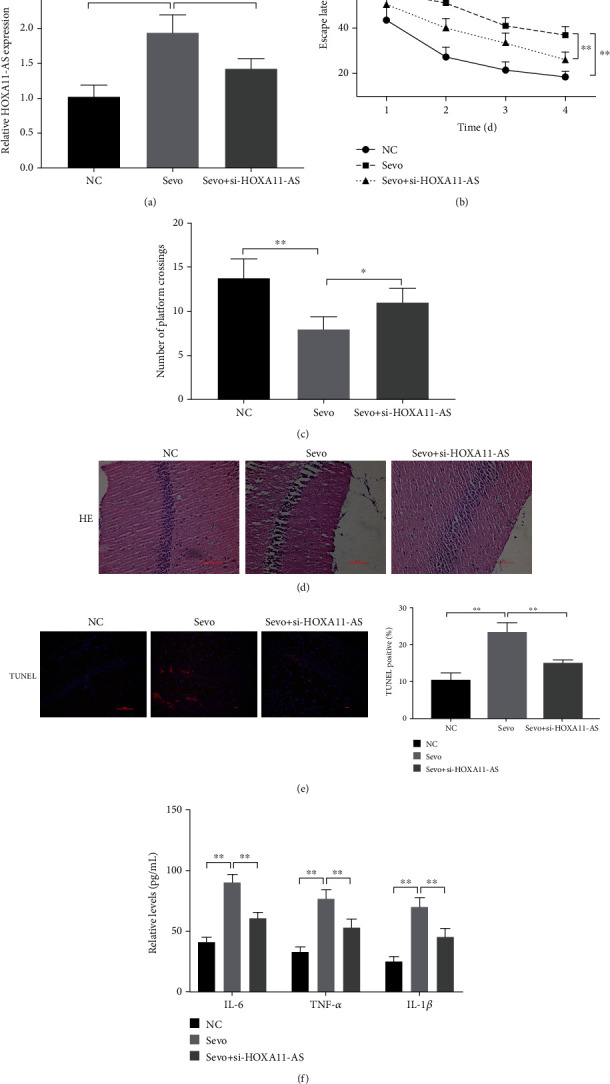
Inhibition of HOXA11-AS alleviates the cognitive impairment caused by sevoflurane in rats. After 3 days of anesthesia, the hippocampal tissue was taken for the following experiments: (a) RT-qPCR detection of HOXA11-AS expression in rat hippocampus. (b, c): MWM test to detect escape latency and the number of platform crossings. (d) HE staining to detect rat hippocampal tissue pathological phenomena. (e) TUNEL staining to detect rat hippocampal tissue nerves apoptosis. (f): ELISA detects inflammatory factor (IL-6, TNF-*α*, and IL-1*β*) levels in rat hippocampus. ^∗∗^*P* < 0.01.

RT-qPCR results showed that sevoflurane significantly induced HOXA11-AS expression increase in the hippocampus of rats, and the HOXA11-AS expression level in the si-HOXA11-AS group was lower than sevoflurane group (Figure 1(a)). We chose HOXA11-AS as the key point to study it and its downstream effects on sevoflurane-induced neuronal apoptosis and inflammation.

In the MWM experiment, after treatment with sevoflurane, the escape latency was significantly increased, and the number of platform crossings was significantly reduced. After knockdown of HOXA11-AS, the percentage of time that mice stayed in the target quadrant was significantly reduced, and the number of platform crossings increased (Figures 1(b) and 1(c)). HE staining showed that the neurons in the hippocampus of the control rats were neatly arranged, the nucleus was clear, and the cytoplasm was uniformly stained; the hippocampal neurons in the sevoflurane treatment group were loosely arranged, and the neurons were swollen and degenerated, nuclear pyknosis and cytoplasmic pigmentation; the morphology of hippocampal neurons in the HOXA11-AS group improved (Figure 1(d)). TUNEL staining showed that the neuronal cell apoptosis increased after treatment with sevoflurane, and neuronal cell apoptosis decreased after transfection of si-HOXA11-AS (Figure 1(e)). In addition, inflammatory factor (IL-6, TNF-*α*, and IL-1*β*) levels in the hippocampus of the sevoflurane-treated rats were significantly increased. After transfection of si-HOXA11-AS, inflammatory factor levels decreased (Figure 1(f)). These results indicate that inhibition of HOXA11-AS can reduce sevoflurane-induced neuronal apoptosis and inflammation and improve sevoflurane-induced cognitive impairment.

### 3.2. Inhibition of HOXA11-AS Reduces Neuronal Apoptosis and Inflammation Induced by Sevoflurane In Vitro

**Figure 2:**
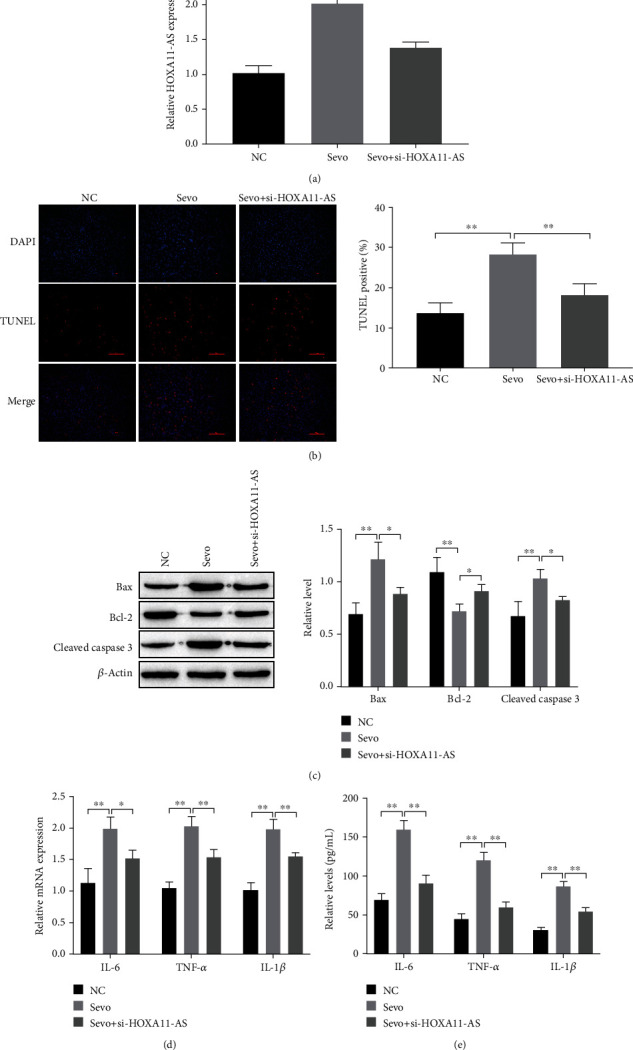
Inhibition of HOXA11-AS alleviates sevoflurane-induced neuronal apoptosis and inflammatory response in vitro. (a, d) RT-qPCR for detection of HOXA11-AS and mRNA expression of inflammatory factors. (b) TUNEL staining to detect neuronal apoptosis. (c) Western blot detects apoptosis-related protein expression. (e) ELISA detects the inflammatory factors levels. ^∗∗^*P* < 0.01.

Since neuronal apoptosis and inflammation are important causes of cognitive impairment in rats caused by sevoflurane [5, 6], we studied the effects of HOXA11-AS on neuronal apoptosis and inflammation induced by sevoflurane in vitro. RT-qPCR results showed that sevoflurane can upregulate HOXA11-AS expression levels, and HOXA11-AS expression levels were significantly reduced after knocking down HOXA11-AS (Figure 2(a)). TUNEL staining and Western blot showed that sevoflurane significantly induced neuronal apoptosis and upregulated apoptosis-inducing factor (Bax and cleaved caspase 3) expression levels, inhibited the apoptosis inhibitor factor Bcl-2 expression levels, and inhibited HOXA11-AS to relieve sevoflurane-induced neuronal apoptosis (Figures 2(b) and 2(c)). RT-qPCR and ELISA, respectively, detected the inflammatory factors levels. The results showed that sevoflurane significantly upregulated inflammatory factor levels in neurons and inhibited HOXA11-AS which alleviates the inflammatory response induced by sevoflurane (Figures 2(d) and 2(e)). It can be seen that inhibiting HOXA11-AS reduces neuronal apoptosis and inflammation caused by sevoflurane in vitro.

### 3.3. miR-98-5p Is the Target of HOXA11-AS

**Figure 3:**
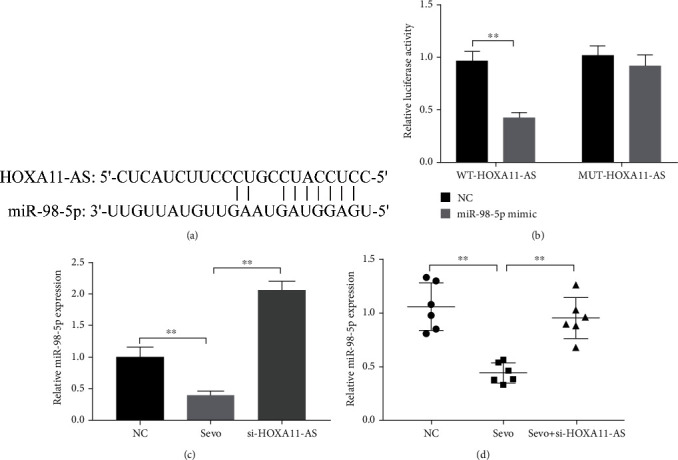
Validation of the correlation between miR-98-5p and HOXA11-AS targeting. (a) Starbase predicts target genes of HOXA11-AS. (b) Dual-luciferase reporter gene verifies the targeting relationship. (c, d) RT-qPCR detects miR-98-5p expression in neuronal cells and rat hippocampus. ^∗∗^*P* < 0.01.

The online tool Starbase predicts that miR-98-5p is a potential target gene of HOXA11-AS (Figure 3(a)). The dual-luciferase reporter gene acknowledged the targeting relationship (Figure 3(b)). Furthermore, we found that miR-98-5p expression levels was upregulated after HOXA11-AS was knocked down (Figure 3(c)). In addition, we also found that sevoflurane inhibited miR-98-5p expression levels (Figure 3(c)). In vivo experiments have shown similar results. RT-qPCR showed that miR-98-5p expression levels in the sevoflurane group were decreased and upregulated after transfection with si-HOXA11-AS (Figure 3(d)). It can be seen that sevoflurane can reduce miR-98-5p expression levels, and HOXA11-AS targets downregulation of miR-98-5p expression.

### 3.4. Effects of miR-98-5p on Sevoflurane-Induced Neuronal Apoptosis and Inflammation In Vitro

**Figure 4:**
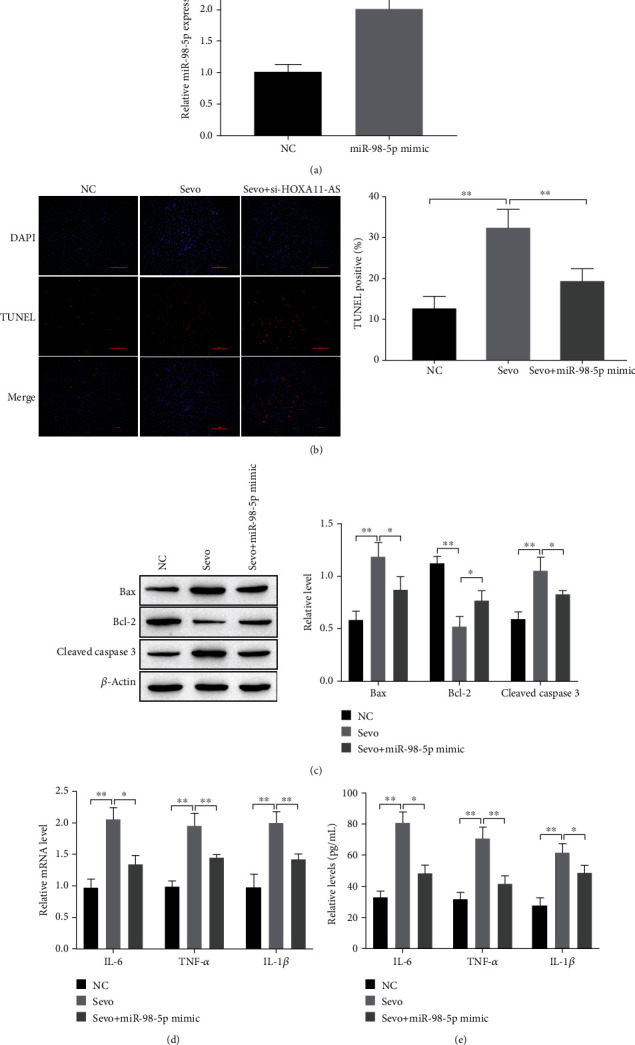
Effects of miR-98-5p on sevoflurane-induced neuronal apoptosis and inflammation in vitro. (a, d) RT-qPCR for detection of HOXA11-AS and mRNA expression of inflammatory factors. (b) TUNEL staining to detect neuronal apoptosis. (c) Western blot detection of apoptosis-related protein expression. (e) ELISA detects the inflammatory factors levels. ^∗∗^*P* < 0.01.

RT-qPCR showed that miR-98-5p expression increased significantly after miR-98-5p mimic was transfected (Figure 4(a)). TUNEL and Western blot showed that miR-98-5p mimic partially inhibited neuronal apoptosis induced by sevoflurane; downregulation of apoptosis inducing factor expression levels, promoted the of apoptosis inhibitor factor expression levels (Figures 4(b) and 4(c)). RT-qPCR and ELISA, respectively, detected the levels of inflammatory factors. Results showed that miR-98-5p mimic partially restored neuronal inflammation induced by sevoflurane (Figures 4(d) and 4(e)). It can be seen that miR-98-5p mimic reduces neuronal apoptosis and inflammation caused by sevoflurane in vitro.

### 3.5. miR-98-5p Targets EphA4

**Figure 5:**
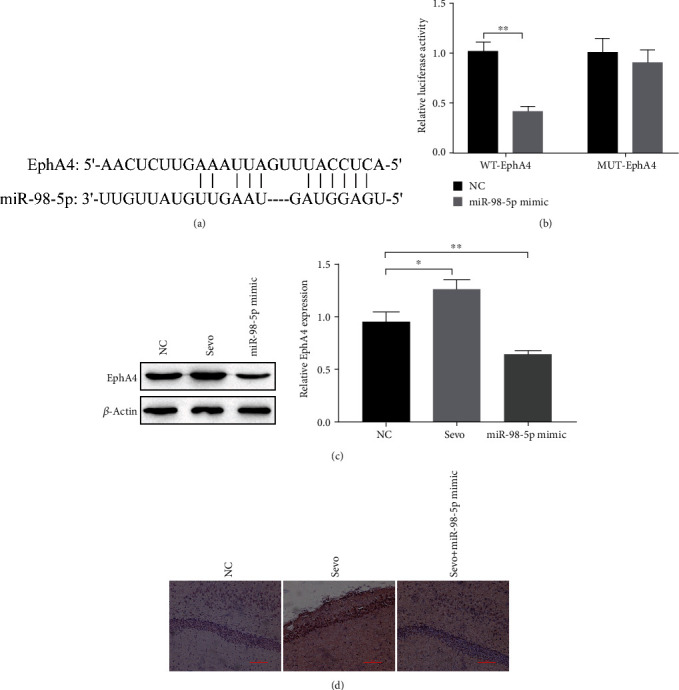
miR-98-5p targets EphA4. (a) Starbase predicts that EphA4 is the target of miR-98-5p. (b) Dual-luciferase reporter gene acknowledged the targeting relationship. (c) Western blot detects EphA4 expression in neurons. (d) Immunohistochemical detection of EphA4 expression in rat hippocampus. ^∗^*P* < 0.05, ^∗∗^*P* < 0.01.

Further, we explored the downstream mechanism of miR-98-5p. An online tool Starbase predicts the target gene of miR-98-5p, and we found that EphA4 is the target gene of miR-98-5p (Figure 5(a)). The dual-luciferase reporter gene acknowledged the targeting relationship (Figure 5(b)). Furthermore, Western blot showed that miR-98-5p mimic significantly downregulated EphA4 expression (Figure 5(c)); however, sevoflurane upregulated EphA4 expression (Figure 5(c)). In vivo experiments have shown similar results. Immunohistochemical showed that EphA4 expression levels were increased by sevoflurane, and EphA4 expression decreased after treatment with miR-98-5p mimic (Figure 5(d)). It can be seen that sevoflurane can increase the expression of EphA4, and treatment with miR-98-5p mimic can lead to downregulation of EphA4 expression.

### 3.6. HOXA11-AS Regulates Sevoflurane-Induced Neuronal Apoptosis and Neuroinflammation through the miR-98-5p/EphA4 Molecular Axis

**Figure 6:**
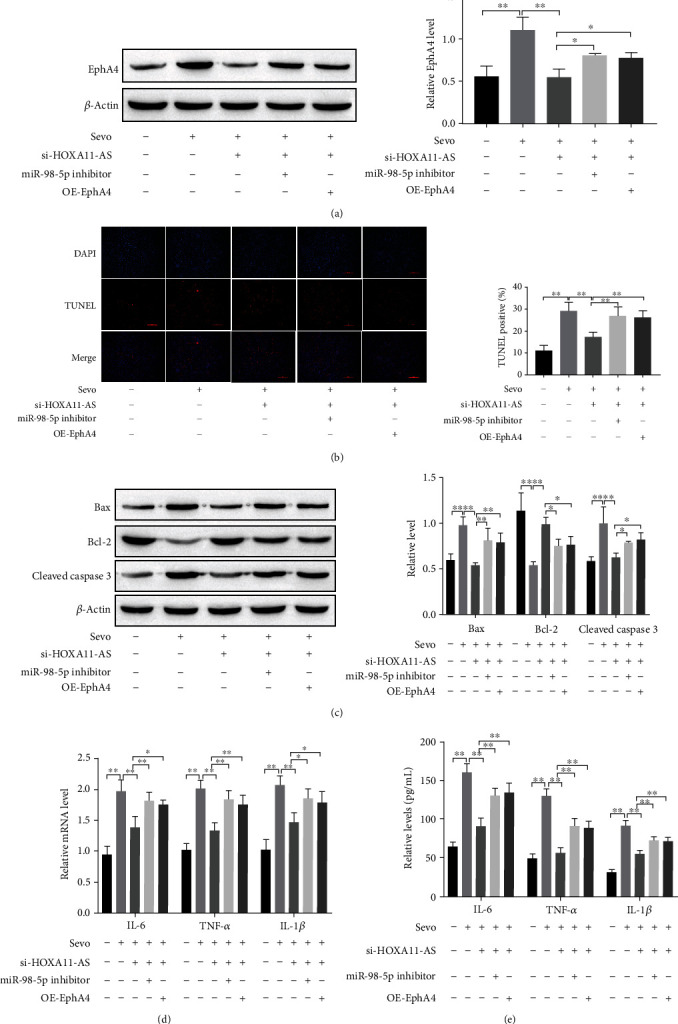
HOXA11-AS regulates sevoflurane-induced neuronal apoptosis and neuroinflammation through the miR-98-5p/EphA4 molecular axis. (a, c) Western blot detection of protein expression in neurons cells. (b) TUNEL staining to detect neuronal apoptosis. (d) RT-qPCR detect the mRNA expression of inflammatory factors. (e) ELISA detects the inflammatory factors levels. ^∗^*P* < 0.05, ^∗∗^*P* < 0.01.

Western blot analysis demonstrated that knockdown of HOXA11-AS reduced the upregulation of EphA4 induced by sevoflurane; both miR-98-5p inhibitor and OE-EphA4 were restored the inhibitory effect of si-HOXA11-AS (Figure 6(a)). TUNEL and Western blot and RT-qPCR and ELISA were used to verify whether HOXA11-AS regulates sevoflurane-induced neuronal apoptosis and neuroinflammation through the miR-98-5p/EphA4 molecular axis. The results show that si-HOXA11-AS has the effect of inhibiting on neuronal apoptosis and neuroinflammation induced by sevoflurane; both miR-98-5p and OE-EphA4 were reverse the inhibitory effect of si-HOXA11-AS on neuroapoptosis and neuroinflammation (Figures 6(b)–6(e)). It can be seen that HOXA11-AS regulates sevoflurane-induced neuronal apoptosis and neuroinflammation through the miR-98-5p/EphA4 molecular axis.

### 3.7. HOXA11-AS Alleviates the Cognitive Impairment Caused by Sevoflurane in Rats through the miR-98-5p/EphA4 Molecular Axis

**Figure 7:**
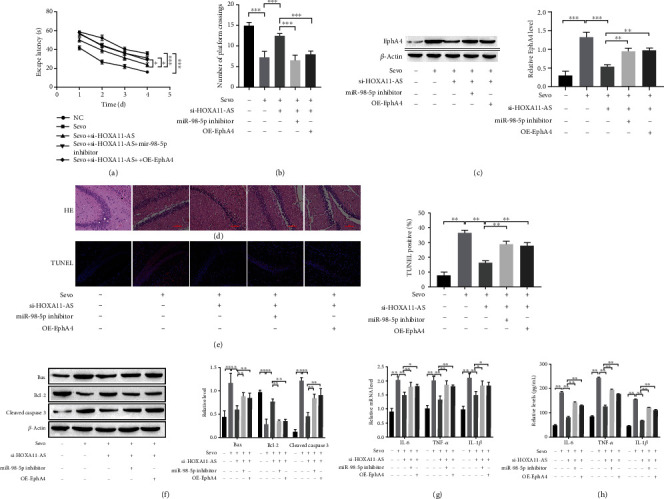
HOXA11-AS alleviates the cognitive impairment caused by sevoflurane in rats through the miR-98-5p/EphA4 molecular axis. After 3 days of anesthesia, the hippocampal tissue was taken for the following experiments: (a, b) MWM test to detect escape latency and the number of platform crossings. (c, f) Western blot detection of protein expression in rat hippocampus. (d) HE staining to detect rat hippocampal tissue pathological phenomena. (e) TUNEL staining to detect rat hippocampal tissue nerves apoptosis. (g) RT-qPCR detect the mRNA expression of inflammatory factors. (h) ELISA detects the inflammatory factors levels in rat hippocampus. ^∗^*P* < 0.05, ^∗∗^*P* < 0.01, ^∗∗∗^*P* < 0.001.

Encouraged by the above results, we further studied HOXA11-AS's regulation of sevoflurane-induced neuronal apoptosis and neuroinflammation in rats at the animal level through miR-98-5p/EphA4 molecular axis. In the MWM experiment, after treatment with sevoflurane, the escape latency was significantly increased, and the number of platform crossings was significantly reduced. After knockdown of HOXA11-AS, the percentage of time that rats stayed in the target quadrant was significantly reduced, and the number of platform crossings increased; miR-98-5p inhibitor or overexpression of EphA4 reverses the above results (Figures 7(a) and 7(b)). Western blot analysis demonstrated that knockdown of HOXA11-AS reduced the upregulation of EphA4 induced by sevoflurane in rats; both miR-98-5p inhibitor and OE-EphA4 were restored the inhibitory effect of si-HOXA11-AS (Figure 7(c)). HE staining showed that the neurons in the hippocampus of the control mice were neatly arranged, the nucleus was clear, and the cytoplasm was uniformly stained; the hippocampal neurons in the sevoflurane treatment group were loosely arranged, and the neurons were swollen and degenerated, nuclear pyknosis and cytoplasmic pigmentation; the morphology of hippocampal neurons in the HOXA11-AS group improved; MiR-98-5p inhibitor or overexpression of EphA4 reverses the above results (Figure 7(d)). TUNEL staining showed that the neuronal cell apoptosis increased after treatment with sevoflurane, and neuronal cell apoptosis decreased after transfection of si-HOXA11-AS; miR-98-5p inhibitor or EphA4 overexpression promote neuronal apoptosis in rats (Figure 7(e)). The results of Western blot, RT-qPCR, and ELISA show that si-HOXA11-AS has the effect of inhibiting on neuronal apoptosis and neuroinflammation induced by sevoflurane; both miR-98-5p inhibitor and OE-EphA4 reverse the inhibitory effect of si-HOXA11-AS on neuroapoptosis and neuroinflammation (Figures 7(f)–7(h)). It can be seen that HOXA11-AS regulates sevoflurane-induced neuronal apoptosis and neuroinflammation through the miR-98-5p/EphA4 molecular axis in rats.

## 4. Discussion

In the past 20 years, numerous animal studies have shown that general anesthetic (GA) agents may cause neuronal apoptosis and other neurodegenerative changes in the developing mammalian brain [16]. At the same time, it has also been found that prolonged exposure to multiple anesthetics in newborns may lead to extensive apoptotic neurodegeneration with time- and dose-dependent long-term neurodevelopmental disorders [17]. Sevoflurane is a volatile anesthetic that is well tolerated by inhalation induction. Inhalation anesthetics such as sevoflurane have been shown to induce apoptosis in multiple regions of the mouse brain, including the cerebral cortex and hippocampus [18]. Recently, a number of clinical studies have shown that children who underwent repeated prolonged GA before the age of 4 had worse mental performance than children who did not experience GA [3].

As is well known, lncRNAs are related to many biological processes during development (such as neuronal development, plasticity, disease, and evolution) and are related with sevoflurane [19, 20]. For example, overexpression of Gm15621 [21] or knockdown of Gm43050 [22] both attenuates sevoflurane-induced neuronal apoptosis and inflammatory responses. Wei et al. [23] also found that knocking down NEAT1 improved sevoflurane-induced neurotoxicity through miR-298-5p/SRPK1. Our study found that HOXA11-AS is highly expressed in the human neuron SH-SY5Y cells induced by sevoflurane and the hippocampus of animal models (Figure 1). Knockdown of HOXA11-AS prevents neuronal apoptosis and inflammation caused by sevoflurane treatment (Figure 2).

miRNA can regulate protein expression through mRNA degradation or translational inhibition [24, 25]. They are involved in regulating almost all pathophysiological processes, including growth, development, inflammation, swelling, and neurodegenerative diseases [24]. Multiple miRNAs were dysregulated after exposure to sevoflurane in neonatal rodent models. Which suggests that miRNA may be related to the occurrence and development of sevoflurane induced neurodegenerative diseases. For example, Xiong et al. [26] found that miR-125b-5p was highly expressed in sevoflurane established rat models, and reduction of miR-125b-5p prevented sevoflurane-induced rat hippocampal cell apoptosis and inflammation. Shen et al. [27] reported that miR-211-5p was significantly increased after treatment with sevoflurane in a neonatal rodent model, and knockdown of miR-211-5p reduced neuronal apoptosis. In our study, double-luciferase results indicate that OXA11-AS targets miR-98-5, and miR-98-5p was lowly expressed in sevoflurane induced neuronal cells and in the hippocampal tissues of animal models (Figure 3). Overexpression of miR-98-5p inhibited neuronal apoptosis and inflammatory responses caused by sevoflurane treatment (Figure 4).

Erythropoietin-producing hepatocellular (Eph) tyrosine kinase receptors (RTKs) are membrane-bound proteins, as well as its ligands ephrins (Eph receptor-interacting proteins) [28]. They are important for developmental processes such as cell migration, axon guidance, and spatial organization during CNS development [29]. EphA4, considered as pan-receptor, is a member of the EphA receptor family. It binds to all ephrin ligands extensively; five glycosyl phosphatidyl inositol linked membrane-bound type A ephrins and three transmembrane type A ephrins included. This suggests that EphA4 interacts with type A and type B ephrins to regulate normal and pathophysiological functions. That is, blocking EphA4 may be more effective compared with other Ephs or ephrin [30]. Studies have also found that during the development of the neuronal system, EphA4 plays a crucial role in axon guidance [31, 32] and can also directly act on motor neurons to cause cell death [30]. Current studies have shown that EphA4 inhibitors have neuroprotective effects on Alzheimer's disease, spinal cord injury, and stroke in mouse models [33]. Our study found that double-luciferase results indicate that miR-98-5 targets EphA4; EphA4 is highly expressed in the neuronal cells induced by sevoflurane and the hippocampus of animal models (Figure 5). Knockdown of HOXA11-AS inhibited neuronal apoptosis and inflammation caused by sevoflurane treatment by inhibiting EphA4 expression resulted from miR-98-5p.

In order to further verify that HOXA11-AS regulates sevoflurane-induced neuronal damage and sevoflurane-induced cognitive damage in rats through miR-98-5p/EphA4 molecular axis, we conducted validation experiments at the cellular (Figure 6) and animal levels (Figure 7). The results showed that knockdown of HOXA11-AS inhibited sevoflurane-induced neuronal apoptosis and inhibited secretion of IL-6, TNF-*α*, and IL-1*β*; miR-98-5p inhibitor or overexpression of EphA4 reverses the above results. Animal experiments show that the escape latency was significantly increased, and the number of platform crossings was significantly reduced; neuronal apoptosis and secretion of IL-6, TNF-*α*, and IL-1*β* were increased, in rat hippopus after treatment with sevoflurane. After knockdown of HOXA11-AS, the percentage of time that rat stayed in the target quadrant was significantly reduced, and the number of platform crossings increased; it inhibits sevoflurane-induced neuronal apoptosis and secretion of inflammatory cytokines IL-6, TNF-*α*, and IL-1*β*; miR-98-5p inhibitor or overexpression of EphA4 reverses the above results.

With the improvement of experimental technology, scientists have gone deep into the molecular mechanism of neuronal cell damage. For example, the research results of Li et al. show that miR-424 inhibits apoptosis and inflammatory responses induced by sevoflurane through TLR4/MyD88/NF-*κ*B pathway [34]. Li et al. uncovered an inhibitory role of luteolin in sevoflurane-induced neuronal apoptosis and inflammatory response through activation of autophagy arising from upregulation of heme oxygenase-1 (HMOX1), thereby alleviating sevoflurane-induced cognitive impairment in mice [35]. In addition, overexpression of lncRNA Gm43050 alleviates apoptosis and inflammation response induced by sevoflurane treatment by regulating miR-640/ZFP91 [22]. In our study, we discussed and verified that HOXA11-AS regulates sevoflurane-induced neuronal damage and sevoflurane-induced cognitive damage in rats through miR-98-5p/EphA4 molecular axis, which provides new opinion for treatment of neuronal cell damage caused by anesthetics. However, this study has not yet discussed other factors affecting the signal pathway, which needs to be further improved in future research.

## Figures and Tables

**Table 1:** Primer sequences.

Target	Sequence (F: forward primer, R: reversed primer)
U6	F: 5′-CTCGCTTCGGCAGCACA-3′
	R: 5′-AACGCTTCACGAATTTGCGT-3′
*β*-Actin	R: 5′-GGGAAATCGTGCGTGACATTAAG-3′
	R: 5′-TGTGTTGGCGTACAGGTCTTTG-3′
HOXA11-AS	F: 5′-GCTCTCATTCACGGTCACTTC-3′
	R: 5′-TCTGGCTCTGAGGAGTCACT-3′
miR-98-5p	F: 5′-TGAGGTAGTAGTTTGTGCTGTT-3′
	R: 5′-GCGAGCACAGAATTAATACGAC-3′
IL-1*β*	F: 5′-CCACCACTACAGCAAGGG-3′
	R: 5′-GAACTGGGCAGACTCAAA-3′
TNF-*α*	F: 5′-AGTGACAAGCCTGTAGCCC-3′
	R: 5′-GCAATGATCCCAAAGTAGACC-3′
IL-6	F: 5′-CCTTCGGTCCAGTTGCCCTTCT-3′
	R: 5′-GCATTTGTGGTTGGGTCA-3′

## Data Availability

The datasets used and/or analyzed during the current study are available from the corresponding author upon reasonable request (raw data link: https://www.jianguoyun.com/p/DTTLg4MQgKrPChj-0c0EIAA).
